# Survey of self-assessed preparedness for clinical practice in one Croatian medical school

**DOI:** 10.1186/1756-0500-2-152

**Published:** 2009-07-27

**Authors:** Katarina Bojanić, Gregory J Schears, Darrell R Schroeder, Sarah M Jenkins, David O Warner, Juraj Sprung

**Affiliations:** 1 Department of Obstetrics, Section of Neonatology, Merkur Clinical Hospital, Zagreb, Croatia; 2 Department of Anesthesiology, Mayo Medical School, College of Medicine, Mayo Clinic, Rochester, MN, USA; 3 Department of Health Sciences (Statistics), Mayo Clinic, Rochester, MN, USA

## Abstract

**Background:**

The Croatian higher education system is in the process of reforming its medical curricula to comply with European Union standards. We conducted a survey of students enrolled at the University of Zagreb (Croatia) asking them to rate their perception of preparedness for clinical practice prior to initiation of the reform process. The purpose of the survey was to identify self-perceived deficiencies in education and to establish a reference point for the later assessment of ongoing educational reform.

**Findings:**

One-hundred and forty seven (N = 147) graduates reported the levels of perceived preparedness on 30 items grouped into 8 educational domains. Main domains were: understanding science, practical skills/patient management, holistic care, prevention, interpersonal skills, confidence/coping skills, collaboration, and self-directed learning. For each item, graduates self assessed their preparedness on a scale ranging from 1 to 4, with 1 = "Very inadequate", 2 = "Somewhat inadequate", 3 = "Somewhat adequate", and 4 = "Very adequate". In 7 out of 8 domains the achieved median score was ≥ 3. Students expressed low confidence (defined when ≥ 25% of respondents supplied a rating for the survey question as: "very inadequate" or "somewhat inadequate") with interpersonal skills (discussing terminal disease, counseling distraught patients, balancing professional and personal life), and in performing certain basic semi-invasive or invasive procedures.

**Conclusion:**

Zagreb medical graduates identified several deficiencies within educational domains required for standard clinical practice. Ongoing educational efforts need to be directed towards the correction of these deficiencies in order to achieve standards required by the European Union.

## Introduction

The main role of medical schools is to prepare students to function as competent physicians. Preparedness for practice is typically assessed and licensed *via *medical board (licensing) examinations. While these instruments aim to evaluate mastery of requisite medical knowledge, they are less able to assess other competencies important to practice such as communication, proficiency in physical examination, ability to provide holistic care, and appropriate technical skills. These competencies are more difficult to measure, and the extent to which medical schools provide these skills to their graduates has seldom been studied[1]. In an effort to improve medical education and assessment of these skills, Objective Structured Clinical Examinations (OSCEs) have been introduced in both the United States and Canada which evaluate medical knowledge, clinical and communication skills at both graduate and postgraduate levels[2].

Self-evaluation, a form of assessment which tests an individual's subjective feelings regarding preparedness for tasks, has been used for formative assessment [3-5]. Some authors [5,6] reported a reasonable agreement between trainees' estimates of their own abilities and their actual performance, while others did not [7-12]. While self-perceived assessment of competence may not always correlate with performance, particularly in those who perform poorly,[13] one recognized educational goal is improving students' belief that he or she is capable of attaining a certain goal ("self-efficacy")[14]. Therefore, it is important that medical educators identify areas of educational concern and then use this information to improve students' confidence[15]. The assessment of self-efficacy for clinical skills is increasingly relied upon by the curricular managers, alongside the measurement of observed competence to evaluate curricular success and guide teaching activities[16,17].

The Croatian system of medical education is presently undergoing major restructuring to harmonize students' performance with that in European Union.[18] The final document outlining these changes, denoted the Bologna Process Principles,[19,20] was signed by Croatia in 2005. The fundamental purpose of Bologna reform is to place the student at the center of the learning process and improve the quality of academic programs. The primary aim of this initiative in the area of medical education is to improve curricular content and to increase direct student participation in patient care. In Croatia, this initiative substantially changed the method by which education is delivered. For example, preclinical education (first three years) has been changed from longitudinal (spread out form) to block/modular form. During subsequent clinical years (last 3 years of medical school), students receive lectures in block/modular form and receive clinical training in clinical skill labs and on patient wards. An emphasis is placed on small size teaching groups which increases interaction with teachers.

Croatian medical schools provided traditional teaching until 2006; after that time Bologna educational reform was initiated. Presently, we have no information whether Croatian graduates feel ready to work as junior doctors. Availability of this information could help in designing new educational curricula. The purpose of this study was to assess the perception of the graduates of medical school in Zagreb, Croatia, regarding their readiness for clinical practice, and to establish a baseline for comparison once full implementation of Bologna education initiative is accomplished in the year 2012.

## Methods

### Setting and Study Design

In Croatia, the average age of the first year medical student is 18 years, and the education lasts 6 years. During the first 3 years, students take preclinical courses, which are structured longitudinally with a final exam after completion of the course. During the last 3 years, students are involved with clinical rotations. In the third year of medical school, students became gradually involved with patient care. Clinical rotations combine lecture-based education followed by short sessions (1–2 hours) on wards where they acquire practical skills working with patients. Therefore, Zagreb students lack longer patient contact that include follow-up from admission to discharge. During the ward sessions, students practice taking a medical history, performing a clinical exam, and at the end of the session the findings are discussed with their assigned physician/professor. Rarely do they perform simple surgical procedures, venipuncture, placement of urinary catheters, etc., and these interventions are mostly observed while performed by either a physician or ancillary staff.

To examine how Zagreb medical school graduating students perceive their preparedness to work as young doctors, we administered an anonymous paper survey entitled "Preparedness for Hospital Practice" [21] within a month before graduation in September 2006. Approval for administration of this survey was obtained from the Ethic Committee (Medical School Zagreb, Croatia).

### Survey Instrument "Preparedness for Hospital Practice"

The survey assesses the subjective feelings of the graduates regarding their medical school educational achievements. This survey was originally designed and validated by Hill et al.[21] Questions are arranged in 8 domains of educational goals that include 1) understanding science, 2) practical skills and patient management, 3) holistic care (comprehensive care, which considers the physical, emotional, social, economic, and spiritual needs), 4) prevention, 5) interpersonal skills, 6) confidence and coping skills, 7) collaboration, and 8) self-directed learning (see details in Table 1).

**Table 1:** Survey items by domain: "My medical education prepared me to.."^a^

**Item**	**Description**
	**Domain 1: *Understanding Science***
1.	Understand the cellular basis of disease
2.	Apply principles of basic science to clinical conditions
3.	Justify drug uses on the basis of their mechanisms of action
4.	Select drugs on the basis of their costs, risks and benefits

	**Domain 2: *Practical Skills and Patient Management***
5.	Record clinical data systematically
6.	Carry out an efficient physical examination
7.	Carry out basic ward procedures
	a. *Recording blood pressure*
	*b. Inserting an intravenous line*
	*c. Digitorectal exam*
	*d. Inserting a urinary catheter*
8.	Carry out basic surgical procedures (eg, suturing)
9.	Handle medical emergencies (eg, infarction, stroke, epilepsy status...)

	**Domain 3: *Holistic Care***
10.	Evaluate the impact of family factors on illness
11.	Understand the interaction of social factors with disease (eg, poverty, unemployment)
12.	Appreciate the importance of a patient's cultural/ethnical and religious background

	**Domain 4: *Prevention***
13.	Take a drug and alcohol history with an initial consultation
14.	Encourage patients to improve their health habits (e.g., unhealthy food, obesity, smoking...)
15.	Provide education to patients and families about prevention of disease

	**Domain 5: *Interpersonal Skills***
16.	Feel competent to tell a patient that they have a terminal illness
17.	Deal with dying patients and their family
18.	Feel competent to counsel a distraught patient

	**Domain 6: *Confidence/Coping Skills***
19.	Cope with stress caused by my profession
20.	Balance my work and personal life
21.	Remain calm in difficult situations
22.	Approach confidently senior staff for help in interpreting investigations

	**Domain 7: *Collaboration***
23.	Be sensitive to the needs of nursing staff
24.	Be able to coordinate a comprehensive patient management plan with other specialists and allied health professionals (eg, physiotherapists)
25.	Appreciate the importance of group dynamics when working within a team environment

	**Domain 8: *Self-Directed Learning***
26.	Invest time in developing my knowledge and skills
27.	Keep up to date with medicine

^a ^Summary of responses (%) within each item are presented in Figure 1.

### Analyses

Each *item *on the survey had a response scale to a question beginning with the phrase "my medical education prepared me to..." rated from 1 to 4, with 1 = "Very inadequately", 2 = "Somewhat inadequately", 3 = "Somewhat adequately", and 4 = "Very adequately". Domain scores were calculated for each respondent by taking the mean response (scale 1–4) for the set of items within each domain. An overall score was computed as well by taking the mean response over all of the items. In all cases, higher scores indicate higher feelings of adequacy in preparedness within the given domain. The domain scores were summarized using median and interquartile range (IQR). To supplement the presentation of domain scores, the *item *level responses were summarized graphically. For this summary, the two response options for inadequacy ("very inadequate" + "somewhat inadequate") were combined into a single "inadequate" category. For interpretation purposes, performance was considered to be "deficient" for items rated as "inadequate" by ≥ 25% of students. The cut-off of 25% was arbitrarily selected as a realistic expectation for evaluating achievement and was determined prior to evaluating the results.

## Results

The survey response rate was 61% (147/240), and 60% of respondents were females. Table 2 shows how graduates rated overall satisfaction with their education. The majority of graduates (71%) were only "somewhat satisfied", while similar proportions were "fully satisfied" (12.9%) or "dissatisfied" (15.7%). The primary educators during hospital rotations in Zagreb were 'multiple educators' which include professors, specialists, and/or residents.

**Table 2:** Overall satisfaction with medical education and role of primary educator

**Characteristic**	**Zagreb** N = 147
***Satisfaction with education***	**N (%)**
Strongly satisfied	19 (12.9)
Somewhat satisfied	105 (71.4)
Somewhat/strongly dissatisfied	23 (15.7)
***Primary educator during hospital rounds***^†^	
I worked mostly on my own	0 (0)
Intern	4 (2.7)
Resident/fellow	0 (0)
Specialist/Consultant	43 (29.5)
Multiple staff^#^	99 (67.8)

^†^Missing 1 response; ^#^Interaction with multiple educators during rotations; N, number

**Figure 1:**
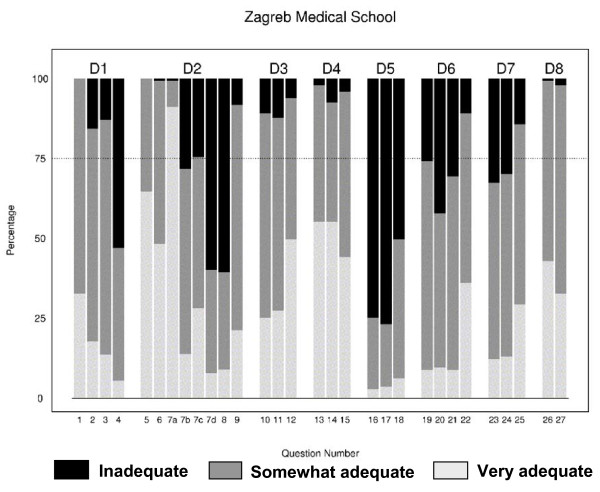
**Response percentages for each *item *within the 8 surveyed domains**. For simplicity of graphical presentation, we combined two response options for inadequacy ("Somewhat inadequate" and "Very inadequate") into a single "inadequate" (black shaded) category. Achievement for a given item was considered deficient if ≥ 25% respondents rated the *item *as "inadequate". A reference line at 75% is provided to help distinguish "inadequate" from "adequate items". Domain titles and *item *details are provided in Table 1.

In considering responses regarding confidence in major educational domains, the median domain score was ≥ 3.0 ("Somewhat adequate") for 7 of 8 domains, while 1 domain (interpersonal skills) had a median score of 2.0 ("Somewhat inadequate") (Table 3). Only the score that describes confidence within the preventive medicine area achieved responses close to the "very adequate" level. Results for all item level responses are graphically shown in Figure 1. For each item, we considered the response as inadequate (shown as black shaded area in histograms) when at least 25% of students rated the item as "Somewhat inadequate/Very inadequate"). Using these criteria, Zagreb graduates had a relatively high number of deficient items (12/27, 44%). Within the first domain (D1, Table 1, Figure 1) selection of drugs in regard to their cost, risks and benefits was a deficient item. Within D2 domain (D2, Table 1, Figure 1) basic surgical skills and inserting urinary catheters were deficient items. All other deficiencies are based on certain aspects of interpersonal skills such as dealing with patients, nursing staff, or balancing work and personal life (D5 domain: items 16–18, D6 domain: items 20 and 21, D7 domain: items 23 and 34, Table 1, and Figure 1).

**Table 3:** Main domain scores for Zagreb Medical School graduates

**Main Domain**	**Zagreb (N = 147)**
	**Median (25^th^, 75^th^)**

1. Understanding Science	3.00 (2.75, 3.25)

2. Practical Skills & Patient Management	3.00 (2.75, 3.38)

3. Holistic Care	3.33 (3.00, 3.67)

4. Prevention	3.67 (3.00, 4.00)

5. Interpersonal Skills	2.00 (2.00, 2.67)

6. Confidence/Coping Skills	3.00 (2.50, 3.25)

7. Collaboration	3.00 (2.67, 3.33)

8. Self-Directed Learning	3.00 (3.00, 4.00)

**Overall score**	3.00 (2.77, 3.23)

*Score range from 1 to 4 for each item (see Methods). Higher scores indicate feelings of more adequate learning while low scores indicate feelings of inadequate learning.

## Discussion

We report survey results from the last generation of Medical School in Zagreb (graduating year 2006) who completed their medical education under traditional teaching, before the new educational initiative was introduced. The main finding of our survey is that graduates expressed relatively low confidence in several competences regarding their preparedness for their upcoming internship. Graduates reported low scores regarding skills in discussing terminal disease, dealing with a dying patient, and counseling distraught patients. Direct communication with patients and conveying important information regarding seriousness of their illness is not a part of the educational curricula during the 6 year medical training in Croatia. Terminally ill patients in Croatia are rarely informed by the physician regarding their poor prognosis, and more typically the "bad news" is communicated first to the family members, and later, depending on circumstances, to the patient. Interestingly, several other deficiencies identified amongst Zagreb graduates are also related within domains of interpersonal skills such as dealing with nursing issues or issues related to balancing work and personal life.

Another apparent deficiency was in the domain of basic clinical skills. Zagreb graduates were confident in elementary clinical skills such as measuring blood pressure, but they felt less prepared in performing simple procedures (such as minor surgical interventions, venipuncture, etc.) or placement of urinary catheters. Measuring of the blood pressure represents the part of the initial patient examination (along with chest auscultation, and determination of pulse rate), and Zagreb medical students are routinely involved in this process, while the procedures such as insertion of urinary catheters, intravenous lines etc., are more frequently observed than performed. These finding are not unique for Croatian medical students. Similar findings were identified in other Western medical programs, [22] and despite improvements in education over the last decade, they continue to persist.[8] A recent report from Britain demonstrated that their interns showed low preparedness in treating minor injuries,[23] and this was not different from an earlier British report which showed that the undergraduate preparation was deficient in some practical procedures, common clinical conditions, and communication skills [24]. Finally, two categories indicated by Zagreb graduates as deficient were lack of confidence when faced with stressful situations and coordination of complex patient management situations. This is not surprising as these types of competences are usually acquired during postgraduate training.

Several factors may be postulated as responsible for the lower levels of self-perceived preparedness in some educational domains among Zagreb graduates. First, Zagreb students participate less in direct patient interaction, clinical decision-making and conducting supervised diagnostic and therapeutic procedures compared to students in Western European or United States educational systems (personal experience JS, KB). In these systems, students take night calls, conduct supervised patient admissions, and follow the patients throughout their hospital stay which may effectively increase knowledge and subsequent confidence. In Zagreb, students observe rather than perform invasive clinical procedures, and this lack of participation may reflect in lower confidence. Second, students in Zagreb acquire clinical skills largely from multiple educators (specialists, professors), while in Western medical educational systems, students receive the majority of clinical knowledge from working with residents and fellows. It is likely that more intense collaborative interaction between medical students and residents, in which workload is shared, has an impact on the education. Third, one of the disadvantages related to Croatian higher education may be associated with the lower financial compensation for physicians, which may affect motivation to teach. Finally, limited personnel resources and burdens on clinicians to complete their clinical duties simultaneously with teaching may diminish the motivation to invest time with students.

Among surgical residents, Kwasnik et al.[25] showed a good correlation between self-evaluation scores and specific clinical performance (knowledge, clinical judgment, and technical ability). At the same time, identification of the level of confidence [15] in specific clinical domains represents an important educational tool which may be used to directly target deficient areas.[16,17]

Ongoing reform in Croatian medical education [19,20] is introducing broader contact between medical student and patients, and more independence (albeit supervised) in performing diagnostic and therapeutic procedures. One of the aims of the educators is to identify areas which students consider to be deficient. This is what our survey has accomplished. Because the present survey assessed the students' belief of ability to function as competent young doctors before the initiation of the reform, our observation may serve as guidance for educational improvements and a baseline for comparison of achievements after their implementation.

### Limitations

When interpreting the results of this investigation there are several potential limitations. First, we report the findings from a single medical school; therefore, the results cannot be generalized to other medical schools in Croatia. Second, the survey instrument "Preparedness for hospital practice" was originally administered to practicing doctors,[21] and not students, and they were asked to rate their preparedness looking retrospectively at their undergraduate training. In the present study, the survey was administered to graduating medical students in anticipation of their work as junior doctors, and the potential exists that these students have little insight into what would be required for them, since they have no actual work experience. Although this questionnaire was not validated on a student population, we believe our results may help to identify some deficiencies and this information may be used to direct medical education.

## Conclusion

In conclusion, we identified several major areas of clinical competence that could be improved among Zagreb medical students. Since Croatian education is currently undergoing reform, our results may be used to guide these changes as well as provide a point of reference to assess improvements upon completion of the educational reform.

## Abbreviations

OSCEs: Objective Structured Clinical Examinations; QR: interquartile range.

## Competing interests

The authors declare that they have no competing interests.

## Authors' contributions

KB (study design, interpretation and collection of data, and writing the manuscript). DRS, GJS, SMJ (performed the analyses, interpretation of data, and writing the manuscript). DOW, JS (study design, interpretation of data, and writing the manuscript). All authors read and approved the final manuscript.
